# Spiritualism and Insanity

**Published:** 1910-05-07

**Authors:** Dudley Wright

**Affiliations:** Editor *Annals of Psychical Science.* London, W.C.


### Medical Letter-Box.

### "SPIRITUALISM AND INSANITY."

*To the Editor of* The Hospital.

Sir,—I am sure many of your readers will not agree with the sweeping statement of the reviewer of this booklet in your current issue, when he speaks of exposing "the chicanery with which all spiritualism is bound up." It would be folly to deny that there have been fraudulent mediums in the past, and probably there are some to-day, but in nearly every instance the credit of the exposure of those mediums must be given to spiritualists themselves. But one cannot dismiss in the summary manner of your reviewer the unprejudiced and scientific researches of Professor Richet, Professor Morselli, Dr. Maxwell, Dr. Ochorowicz, Professor Lombroso, Sir William Crookes, and many other scientists of repute, who have all certified the veridical character of the phenomena.

110 St. Martin's Lane, Yours faithfully,

London, W.C. Dudley Wright,

April 18, 1910. Editor *Annals of Psychical Science.*

